# Psychiatric Emergency in Children and Adolescents: A Retrospective Study in Parma Local Health Unit

**DOI:** 10.1155/2021/8848387

**Published:** 2021-10-25

**Authors:** Michela Deolmi, Emanuela Claudia Turco, Pietro Pellegrini, Carlo Marchesi, Francesco Pisani

**Affiliations:** ^1^Pediatric Unit, Medicine & Surgery Department, University of Parma, Parma 43126, Italy; ^2^Child Neuropsychiatry Unit, University Hospital of Parma, 43126, Italy; ^3^Department of Mental Health and Pathological Addiction, Azienda USL di Parma, Parma 43126, Italy; ^4^Psychiatry Unit, Medicine & Surgery Department, University of Parma, Parma 43126, Italy; ^5^Child Neuropsychiatry Unit, Medicine & Surgery Department, University of Parma, Parma 43126, Italy

## Abstract

The mental health care system in Italy is based on Law 180/70 which leaves great regional autonomy about the management of adolescent patients suffering from psychiatric diseases. The aim of this study is the evaluation of demographic, social, and clinical features of minors admitted to psychiatric wards, as starting point to improve individualized services for them. Data about all under 18s consecutively admitted to Parma's psychiatric wards from 2013 to 2015 were retrospectively collected from medical records. Diagnoses were classified according to ICD-10 criteria, and statistical analysis was performed using SPSS statistical software (IBM SPSS Statistics 22.0) for Windows. Clinical samples include 51 cases, 30 males (mean age: 15.5 years, ranging from 12 to 17 years) and 21 females (mean age: 15.9, ranging from 14 to 17 years). The most frequent diagnosis is conduct disorder (39.2%), with higher prevalence among males. Following this, 23.5% of the patients present comorbidity issues and 9.8% suffer from personality disorders, which is more frequent among females. High percentages of foreigners (31.4%), adopted minors (15.7%), and drug users (40%) are reported. Furthermore, data reveal that unprotective family environment, registered in 80.4% of cases, plays an important role as risk factor for the development of mental disease, readmissions in psychiatric wards, and discharge to residential facilities. Readmissions, as well as compulsory treatments (11 cases), are mainly required in case of conduct disorders and comorbidity diagnosis. Lastly, in contrast with the situation before hospital admission, most patients (63.3%) are discharged and sent to community residential facilities. Findings can be useful to improve the management of psychiatric emergencies in minors, focusing on their specific needs, such as conduct disorders and substance abuse, and to face emerging challenges, for example, mental health disease associated with the growing phenomenon of immigration.

## 1. Introduction

Psychiatric emergencies in children and adolescents have been challenging the mental health system with an increasing number of requests over the years, and a systematic review including studies from 27 countries shows that the worldwide prevalence of mental health disease in children and adolescents is 13.4% [1]. In fact, in the United States, the percentage of medical examinations in minors due to psychiatric disorders in the Emergency Room has grown up to 47% in ten years [2] and a further growth is expected in the future [3], particularly in Italy, where a 38% increase in psychiatric medical examinations has been registered in 2004 with respect to 2001 [4]. Many studies report that the onset of mental disease usually occurs in childhood, mostly with behaviour and anxiety disorders, or in adolescence, with high prevalence of anxiety, mood disorders, and substance abuse. Early diagnosis of this mental disease can improve their pathologic course, in terms of recurrences, readmission, and in some cases of endurance [5]. Therefore, paediatric mental health care settings need to be improved for children and adolescents suffering from psychiatric diseases. In Italy, Law 180/78 only provides general guidelines about mental health service organization and does not indicate how to deal with psychiatric emergency in minors. Moreover, the policy about hospital admission in psychiatric wards, as well as literature, is focused on adult patients and there is little about minors' management, with no shared guidelines. According to the American Psychiatric Association, minors' hospitalization is necessary in case of psychiatric diseases' acute phase, posttraumatic stress disorders, suicide attempts, intoxication or withdrawal in patients suffering from addiction disorders, major depressive disorder, disabling conditions in compulsive and anxiety disorders, severe psychosomatic disorders, life-threatening eating disorders, aggressive behaviour and state of agitation in chronic conditions, maladaptive behaviour in personality disorders, and in every case of debut psychiatric diseases [6]. Since the beginning of 2000s, childhood and adolescence psychiatric services have been improved, but despite this, a minor presenting an acute or enduring mental health problem is often admitted in adult psychiatry settings. However, during childhood and even more during adolescence, a lot of challenges face the creation of a balanced mental health status. Hence, psychiatric services should promote mental development as healthy as possible [7, 8], creating a suitable setting where minors' treatment can be carried out [9, 10].

The aim of this study is to collect data about children and adolescents that have been hospitalized due to psychiatric emergencies for better understanding of their needs and to create more suitable settings for their admission, differentiating them from the adults' ones.

The primary objective of this study is the evaluation of demographic, social, and clinical characteristics of inpatients underage 18 admitted to psychiatric wards in Parma's Local Health Unit, as a starting point to improve customized services for them. Further aims are to investigate the association of the main risk factors with the rate of hospitalization and number of readmissions and the comparison between involuntary and voluntary admitted inpatients, as well as to assess which conditions and personal characteristics are more frequently associated with compulsory treatment. Lastly, the study compares minors living in their family of origin and those living in outpatient residential facilities before and after the hospitalization period.

## 2. Materials and Methods

Clinical samples include minors (age ranging from 12 to 17 years) admitted to Parma Local Health Unit general hospital psychiatric wards, including SPOI (Psychiatric Intensive Care Unit) and SPDC (Diagnosis and Treatment Psychiatric Services) in 2013, 2014, and 2015. Each patient was evaluated through a complete medical history and clinical examination. Data were collected retrospectively from medical records, provided by the archive of each ward and therefore comprehensive of all patients that fulfill the previous criteria. Patients were divided according to admission year (2013, 2014, or 2015).

### 2.1. Measures

A database was created through the software IBM SPSS Statistics 22.0, considering the minors' main demographic information such as age, gender, place of living, birthplace, and possession of Italian citizenship when born abroad. Moreover, main diagnosis, the need for compulsory admission, length of stay in the ward, number of readmissions, and type of discharge were collected.

As indicators of the minor's well-being and risk factors for his mental health, family history and familial environment, if the minor was adopted and at which age, substance abuse, education, and employment were considered. Familial environment was classified as protective when no conflicts exceeding the normal misunderstandings that can take place in a family were reported on medical records and when the family structure was composed by both parents, whether separated or not, taking care about the minor and his mental health. On the other hand, familial context was defined unprotective when

- stressful events such as families broke up, parent's or sibling's death, and severe parent's mental health diseases affected the familial balance
- parents or siblings were suspected and/or jailed to have abused the minor
- the minor was abandoned by one or both parents
- stifling familial atmosphere and conflicts with parents were severe enough to trigger the mental health alteration in the minor or to require social service intervention to separate the patient from his family
- parental responsibility was revoked by the Juvenile Court

Moreover, diagnosis was classified according to the 10^th^ revision of the International and Statistical Classification of Diseases and Related Health Problems (ICD-10), which is the main international diagnostic tool, recognized by the World Health Organization [11]. In addition to this, autism and comorbidity were considered as separated categories.

### 2.2. Data Analysis

Statistical analysis was performed by IBM Statistical Package for the Social Sciences version 22.0 for Windows program (IBM-SPSS Statistic 22). Variables were divided into continuous or nominal, and descriptive statistics were mainly run. After that, Pearson's chi-square test was performed to calculate statistical significance (*p* values under 0.05 were interpreted as significant), and for independent variables, *t*-tests were applied.

### 2.3. Ethical Approval

This study has been approved by the Regional Ethical Committee (Prot. N. 24486; date: 10/06/2019), and it has been performed in accordance with the ethical standards laid down in the 1964 Declaration of Helsinki and its later amendments.

## 3. Results

### 3.1. Demographic and Social Features

Clinical samples include 51 cases, 30 males (average age: 15.5 years, ranging from 12 to 17 years) and 21 females (average age: 15.9, ranging from 14 to 17 years).

The percentage of minors born abroad is 31.4%. Adopted minors are almost 16%.

### 3.2. Rate of Diagnosis, Readmission, and Length of Stay

**Figure 1:**
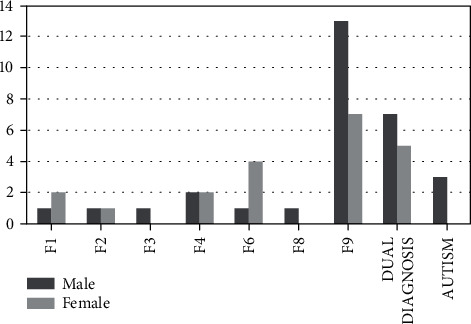
Correlation between gender and diagnosis. Conduct disorder is more frequent in males, while personality disorders are more frequent in females. F1: mental and behavioural disorders due to psychoactive substance use; F2: schizophrenia and schizotypal and delusional disorders; F3: mood disorders; F4: neurotic, stress-related, and somatoform disorders; F6: disorders of adult personality and behaviour; F8: disorders of psychological development; F9: behavioural and emotional disorders with onset usually occurring in childhood and adolescence, including conduct disorder.

In the studied population, the most frequent diagnosis is conduct disorder (39.2%), with higher prevalence among males without difference between native and foreigners.  Figure 1 shows correlations between gender and diagnosis. In particular, conduct disorder is more frequent among males, while personality disorders show higher prevalence in females.

Moreover, the higher rate of readmission is observed in patients with conduct disorders or with comorbidity without difference among voluntary and involuntary admitted patients.

Length of stay is about 8 days for each patient, considering the first admission and readmissions.

### 3.3. Risk Factors

#### 3.3.1. Substance Abuse

Substance abuse is one of the main environmental influences on mental health development in early adolescence, when patients come for the first time in contact with alcohol and drugs, usually starting by cannabis consumption (31% of the patients in this study) till the so-called “heavy” drugs, such as cocaine (5.9%) or heroin (3.9%), but also synthetic drugs (MDMA 5.9% and LSD 2%) and medications (5.9% benzodiazepine). 13.7% of the minors can be classified as “poliabuser,” especially the ones diagnosed with comorbidity, while the abuse of only one substance is more common among patients with conduct disorders.

#### 3.3.2. Family

Unprotective family environment, registered in 80.4% of the cases, plays an important role as risk factor for the development of mental disease, readmissions in psychiatric wards (29 versus two cases of readmission in patient with protective family environment), and discharge to residential facilities. This aspect gains statistical significance in minors suffering from conduct disorders (*Z* = −3.292, *p* < 0.001), while it is less frequently assessed in personality disorders, where conflicts with and among parents do not seem to trigger symptoms.

#### 3.3.3. Foreign Origins

Admitted minors born abroad are 33.3%. More specifically, 11.8% of the patients come from European countries other than Italy, 13.7% are born in Africa and 7.8% in Centre or South America.  Table 1 shows their country of origin.

#### 3.3.4. Adoption

Among adopted patients (almost 16%), another risk factor is represented by the age of adoption that is usually high: only in two cases children have been adopted in their first year of life, while other minors were more than four years old when they were adopted (two patients adopted at 4 years of age, one at 5, one at 6, one at 7, and one at 11 years of age).

#### 3.3.5. School and Employment

Moreover, certain aspects, such as if the minor is attending school and if he failed some academic years, can be considered both as risk factors and consequences of the mental disease. The percentage of patients that missed some academic years is 19%, while 17% of the patients dropped out of school. Anyway, only two minors are working, meaning that the major part of them is not able either to continue school or to work. Patients' intelligence quotient has not been reported in medical records.

### 3.4. Prevalence of Involuntary and Voluntary Admission

**Figure 2:**
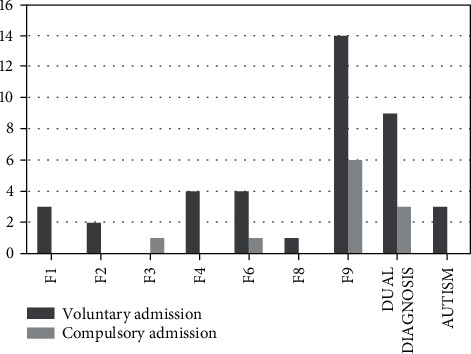
Correlations between compulsory admission, voluntary admission, and diagnosis. Compulsory admission is mainly required in patients suffering from conduct disorder or comorbidity. F1: mental and behavioural disorders due to psychoactive substance use; F2: schizophrenia and schizotypal and delusional disorders; F3: mood disorders; F4: neurotic, stress-related, and somatoform disorders; F6: disorders of adult personality and behaviour; F8: disorders of psychological development; F9: behavioural and emotional disorders with onset usually occurring in childhood and adolescence, including conduct disorder.

Eleven compulsory admissions were registered; hence, 21.6% of the minors were involuntarily treated in Parma's psychiatric wards. The amount of compulsory admissions changes over the years, with a peak in 2014, when six cases (40.0%) were registered compared with only two cases in 2013 (11.1%) and three cases in 2015 (16.7%). Furthermore, involuntary treatment is mainly adopted in patients suffering from conduct disorders or with comorbidity, while only one case of compulsory admission was carried out in minors affected by mood disorder and personality disorder (see Figure 2). None of the patients with other mental diseases required involuntary treatment.

### 3.5. Setting before and after Admission

Lastly, it is important to notice that almost the same percentage of minors used to stay in its family of origin (51.0%) or was hosted in community (49.0%) before admission to psychiatric wards, while there is a significant reduction of minors who live with their parents after the discharge, since they are addressed to a community or a residential facility (63.3%).

**Figure 3:**
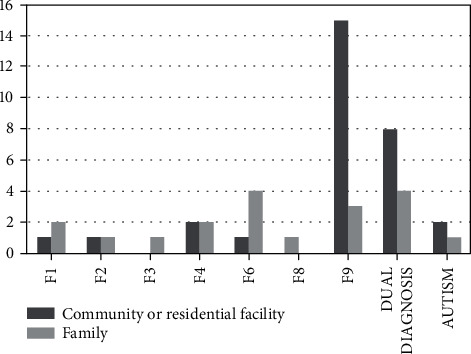
Correlations between place of stay after discharge and diagnosis. The majority of patients with conduct disorder live in a community or residential facility, while minors with personality disorders mainly live with their family of origin. Legend: F1: mental and behavioural disorders due to psychoactive substance use; F2: schizophrenia and schizotypal and delusional disorders; F3: mood disorders; F4: neurotic, stress-related, and somatoform disorders; F6: disorders of adult personality and behaviour; F8: disorders of psychological development; F9: behavioural and emotional disorders with onset usually occurring in childhood and adolescence, including conduct disorder.

Moreover, looking at the correlation between patients with disorders who are suffering and settings in which they live, it is clear that a considerable amount of those who are hosted in residential facilities is composed of minors affected by conduct disorder (46%), while personality disorders often allow patients to keep living with their family (21%). In addition to this, data shows a higher rate of hostile familial contexts among families of minors suffering from conduct disorder than families of minors with personality disorders (*χ*^2^ = 10,864 (1); *p* < 0.001) (see Figure 3). Furthermore, 13 patients hosted in communities or residential facilities needed readmissions in psychiatric ward, while only four cases of minors living with their families were readmitted.

## 4. Discussion

Results show that minor inpatients' mean age is 15.7 years, which is consistent with several studies carried out in other countries such as Turkey, where in a sample of 1080 patients, the mean age was 15.8 years [12], and Finland, where a mean age of 15.1 and 15.9 years was found in two different hospital districts [13]. This indicates that the process of growing up, from childhood to adulthood, is a period of weakness for the mental health status. Adolescents are therefore more vulnerable to environmental factors that can flare up certain character features, leading to the development of a mental disorder which comes out for the first time as an acute episode, requiring a prompt reaction by the mental health system.

Moreover, in this study, a prevalence of male patients has been registered in each considered year, and this is consistent with results of a 5-year retrospective research that took place in Modena, another town located in the same region as Parma, where in a sample of 101 patients, 56 were male [14]. Regarding other European countries, German studies found higher percentage of males in both voluntary (58.7%) and involuntary (53.8%) admitted patients [15]. However, previous research carried out in Turkey and Finland showed a higher presentation of female to psychiatric emergency services (65.8% of Turkish sample and 60%-87.5% in the Finnish study) [12, 13].

As far as clinical features of these patients are concerned, this study reveals that in each considered year, the most frequent diagnosis reported in medical records is conduct disorders, mainly registered in male, but also in female, both in Italians and foreigners, whether voluntary admitted or not. This result is consistent with Finnish, Turkish, and German research that consider minors in psychiatric emergency setting [12, 13, 15]. In addition, this study points out that comorbidity is often associated with admission age older than the average (17 years) because during the process of growing up the onset of a new psychiatric condition in people already affected by another one is not rare, which can be both cause and consequence of the previous mental health status and share with the latter the same biologic and environmental background.

Additionally, this study points out that emerging challenges in Parma's mental health services are psychiatric disease in foreigners, because immigrants and their children have been recently increasing in number among our population [16, 17] and therefore their possibility to access the hospital facilities and the mental health system has grown up [18, 19]. Considering that the percentage of resident foreigners in Italy is 8.2% and 10% in Emilia-Romagna region [20], while this study indicates that the 31.4% of the minors admitted to the psychiatric wards were not born in Italy, it is easy to understand that being a foreigner or an immigrant is an important predictor for the development of acute mental disease [21, 22].

Difficulties in integration in a new country, language barriers, different ethnic backgrounds, and low capacity to reach the mental health services before the disease is full-blown require an emergency approach and explain this high percentage [23, 24]. Also, minors that were born in Italy but with foreign parents show higher percentage of mental disease, which can be explained through the consideration that their family represents for them a cultural, religious, and linguistic system of values different from the one of our society and their peers, which can lead to hostilities in family and difficulties greater than the ones already challenging adolescents, in the process of shaping their personality.

But most of all, negative family environment, according to this study and in line with several other research, is the main risk factor for any mental disease, suggesting the important role played by adults in minors' mental health status. 80.4% of the minors admitted to Parma's psychiatric wards did not grow up in a suitable family environment, especially the ones suffering from conduct disorders. Being abandoned by a parent, being physically or sexually abused, or bereavement in the family is a severe stressful event which can trigger an acute bout of psychiatric symptoms in minors [25, 26]. In addition, a hostile atmosphere with inadequate care of minor's needs and poor quality in relationships that accompanies the most important stages of minor's process of growing up is the most suitable condition for the development of a severe and chronic mental disease with acute episodes that cannot be managed in other setting than hospital wards. Furthermore, readmissions are more frequent in those who are not host family anymore. This is because only less severe patients with a protective familial background return to their place after hospital discharge, while several minors are arranged in other settings. These results are consistent with the ones obtained by a retrospective study carried out in the near town of Modena that reveals that 61% of the adolescents admitted to psychiatric wards have been adopted or live in an institution, with immigrant family or with one divorced parent [14]. The same research points out that only 6% of these patients show regular school performance, even if the intellectual level can be considered normal in a major part of them (81%).

Parma's situation looks similar: there is a strong correlation between mental health diseases and personal functioning, which in minors can be assessed mainly through their scholastic results and relationships with peers. Almost 40% of the minors admitted to Parma's psychiatric wards are not attending school anymore and this can be considered because of their psychiatric condition which makes not possible for them to deal with normal tasks required by school, but also a trigger of mental disease when scholastic environment is more stressful than patients can face.

Following this, our sample reveals a high prevalence of adopted children (16%) requiring hospitalization. Literature to compare this data is not available, but it is obvious that the later the adoption is actualised in a child's life, the harder the adjustment period for the minor, because shaping a self-identity and assessing parents' role are influenced by negative and unstable relationships of the child's past. Another important topic when considering risk factors for hospitalization in psychiatric patients is alcohol and drug abuse [27] that influence not only the minor's lifestyle but also his group of peers, since the patient creates himself a negative and self-maintaining environment around him. 39.2% of the patients in this sample can be defined as drug users, with no significant difference in gender; and this is in line with the results of the research that took place in Modena [14]. Moreover, in Parma studied population, consumption of more than one drug is mainly associated with comorbidity or personality disorders, while patients suffering from conduct disorder and mood and schizotypal disorders are usually not poliabusers.

Another topic considered in this research is compulsory admission. In Italy, according to the Law 833/78, involuntary admission is required when an urgent treatment is needed but it cannot be carried out in an outpatient setting and, due to his mental status alteration, the patient refuses it.

Anyway, there are some differences in its regulation among Europe, and also, each region in Italy has some independence concerning the management of the procedure itself; therefore, comparison of involuntary hospitalization rates in different places is difficult. Moreover, literature mainly regards adults and only few studies analyze children and adolescents such as Jendreyschak and colleagues' research, a cross-sectional comparison between minors voluntary and involuntary admitted in German general hospital psychiatric wards [15]. Results about sample's personal characteristics are consistent with this research in which males suffering from conduct disorder or with comorbidity aged from fifteen to seventeen years are prevalent both among voluntary and involuntary admitted patients.

An interesting outcome of this study is the distribution of patients before and after the admission in Parma's psychiatric wards. Only 49% of the minors used to live in residential facilities before the hospitalization, while after the discharge their percentage increases up to 63%. In fact, the admission in psychiatric wards sometimes represents the first contact with mental health services and engenders the overview of minor's situation, inserting him in a wider evaluation, including his social, scholastic, and family environment. Data collected show that when the family environment is negative, the number of minors who do not live with their parents anymore is higher—27 cases—than the number of patients who lives in residential facilities even if their family is considered appropriate—four cases. Once again, conduct disorder seems to be the most challenging disease in minors for Italian health service, requiring long-term and intensive rehabilitation and individualized treatment plans. However, this is true only for minors, because adults in residential setting more often suffer from schizophrenia [28].

To conclude, this study shows that 51 minors older than twelve were admitted to Parma psychiatric wards during the three years considered, even if some research stress the concept that a suitable inpatient setting, different from adult's one, should be provided for children and adolescents [10, 29]. In Finland, for example, where higher priority is given to services customized for minors with respect to other countries in Europe, there is a psychiatric ward for children, because, according to the Finnish law, they cannot be admitted together with adults [30]. Another example is Ireland, where only sixteen- and seventeen-year-old minors can be referred to adult's psychiatric wards [31]. Differences in hospital policy as regards the age of patients that can be admitted allow limited comparison between studies that took place in other countries.

Although we think the objectives of this study could have provided insight on important primary level health care service, the observational time study of three years and the inclusion of only one Local Health Unit are limits that suggest that the conclusion should be interpreted with caution.

## 5. Conclusion

Children and adolescents suffering from psychiatric disease and presenting with emergency conditions are challenging the mental health care system. Hence, efforts should be focused on improving the management of these patients. To provide adequate paediatric psychiatric care, it is necessary to consider data based on the latest research that point out the emerging needs and suggest remodelling inpatient care setting.

## Figures and Tables

**Table 1:** Patients' birth country.

Country	State	Patients
Europe	Italy	34
	Lithuania	1
	Serbia	1
	Russia	1
	Belarus	1
	Germany	1
	Switzerland	1
Africa	Morocco	4
	Ethiopia	1
	Guinea	1
	Ivory Coast	1
America	Brazil	2
	Colombia	1
	Dominican Republic	1

## Data Availability

The clinical and epidemiological data used to support the findings of this study are included within the article.
